# Vitamin B12 Deficiency in Infants With Axial Hypotonia and Psychomotor Regression: Insights From a Moroccan Case Study

**DOI:** 10.7759/cureus.64000

**Published:** 2024-07-06

**Authors:** Inasse Lamouri, Aziza Elouali, Imane Kamaoui, Maria Rkain, Abdeladim Babakhouya

**Affiliations:** 1 Department of Pediatrics, Mohammed VI University Hospital, Faculty of Medicine and Pharmacy of Oujda, Mohammed First University, Oujda, MAR; 2 Department of Radiology, Mohammed VI University Hospital, Faculty of Medicine and Pharmacy of Oujda, Mohammed First University, Oujda, MAR; 3 Department of Pediatrics/Pediatric Gastroenterology, Mohammed VI University Hospital, Faculty of Medicine and Pharmacy of Oujda, Mohammed First University, Oujda, MAR; 4 Department of Pediatrics/Pediatric Cardiology, Mohammed VI University Hospital, Faculty of Medicine and Pharmacy of Oujda, Mohammed First University, Oujda, MAR

**Keywords:** psychomotor regression, breastfeading, infant, pancytopenia, hypotonia

## Abstract

Vitamin B12 deficiency is a rare entity in the pediatric population. It is often of maternal origin in exclusively breast-fed infants. Its clinical manifestations are multiple and unspecific, encompassing hematological problems and neurodevelopmental consequences. Positive diagnosis and early treatment with vitamin B12 supplementation have a rapidly reversible effect on symptoms. Delayed diagnosis, however, may result in irreversible neurological sequelae. We report the case of a six-month-old infant, admitted with hypotonia and psychomotor regression since the age of four months. The laboratory work-up revealed macrocytic anemia with the presence of megakaryocytes and megaloblasts on the myelogram. Vitamin B12 levels were low, and homocysteine levels were high. A maternal workup showed vitamin B12 deficiency in the mother. A brain MRI showed bilateral frontoparietal cortical atrophy. The patient was put on vitamin B12 supplementation with good evolution. The aim of our work is to shed light on the misleading and varied clinical profile of vitamin B12 deficiency in an exclusively breastfed infant, the serious consequences of maternal vitamin B12 deficiency, and the importance of early diagnosis of this condition

## Introduction

Axial hypotonia with or without an impact on psychomotor development may be secondary to a metabolic cause [1]. Vitamin B12 deficiency is one such cause. This is a water-soluble vitamin that comes mainly from animal sources. Vitamin B12 deficiency is a rare entity in the pediatric population and it is important to be aware of it [2,3]. In infants, the main predisposing factor is exclusive breastfeeding by women who are themselves deficient, and/or have an exclusive vegetarian diet [4,5]. There are many suggestive clinical manifestations of varying severity, involving damage to the central nervous system with consequences for hematopoiesis [6]. Positive diagnosis must be made early, as supplementation with this vitamin normalizes hematological and metabolic disturbances and prevents long-term neurological sequelae [5].

We report the case of an infant with severe vitamin B12 deficiency, admitted with hypotonia and psychomotor regression observed by the family since the age of four months.

## Case presentation

The patient was a male infant aged six months and 10 days, delivered via the high route on a scarred uterus, with a normal birth weight of 3700 g, no neonatal distress, no known maternal-fetal infections during pregnancy, and exclusive breastfeeding. At the age of five months, he presented with feeding difficulties and vomiting, indicating an inability to achieve diversification. He also had chronic constipation. The infant had shown psychomotor regression since the age of four months, which worsened over time with a deterioration in general condition.

Clinical examination revealed a pale, hypotonic, hyporeactive infant with delayed stature-weight development. The weight and height were below the second percentile, with a normal voiding. On neurological examination, he showed generalized hypotonia with symmetrical osteotendinous reflexes. Contact was poor, with no smile-response or eye pursuit. He could not hold his head, and the sitting position had not yet been acquired.

The patient underwent an initial biological assessment revealing bicytopenia: macrocytic hypochromic anemia at 7.1, along with neutropenia at 460/uL; white blood cells and platelets were within normal range. As for the biological signs of malnutrition, albumin level was normal at 47 g/l with a correct protein level at 70 g/l. He also had a low prothrombin time (PT) of 57% (exclusively breast-fed infant, not supplemented with vitamin K), and the rest of his liver work-up was normal. A thyroid workup was performed, with no particular findings, and IgA anti-transglutaminase antibodies were normal. A vitamin assay confirmed severe vitamin B12 deficiency at 69 pg/ml, associated with elevated homocysteinemia at 53 umol/l. A maternal workup showed vitamin B12 deficiency at 149 pg/ml (Table 1). In the context of investigating Biermer's disease, the measurement of anti-intrinsic factor antibodies was performed and returned normal.

**Table 1:** Biological values of the patient and vitamin B12 level of his mother

Laboratory parameters	Initial values	Reference ranges
Hemoglobin (g/dl)	7.1	11.1-12.9
Mean corpuscular volume (fL)	90.7	73-79
Mean corpuscular hemoglobin concentration (%)	29	26-30
White blood cell (/ul)	7860	6000-12000
Neutrophil (/ul)	460	1000-8000
Platelets (/ul)	180000	175000-600000
Reticulocyte (/ul)	76800	> 120000
Vitamine B12 (pg/ml)	69	200 -900
Vitamine B9 (ng/ml)	15.2	3-20
Homocysteine (umol/l)	53	<13
Maternal vitamin B12 (pg/ml)	149	200-900
Albumin (g/l)	47	38-47
Protein (g/l)	70	55-75
Prothrombin time (%)	57	70-100

**Figure 1:**
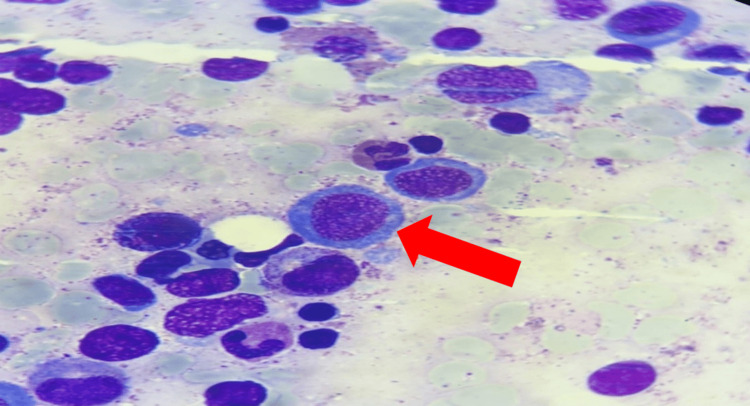
Myelogram showing megaloblastosis Image Credit: Laboratory of Hematology, Mohammed VI University Hospital, Oujda, Morocco

**Figure 2:**
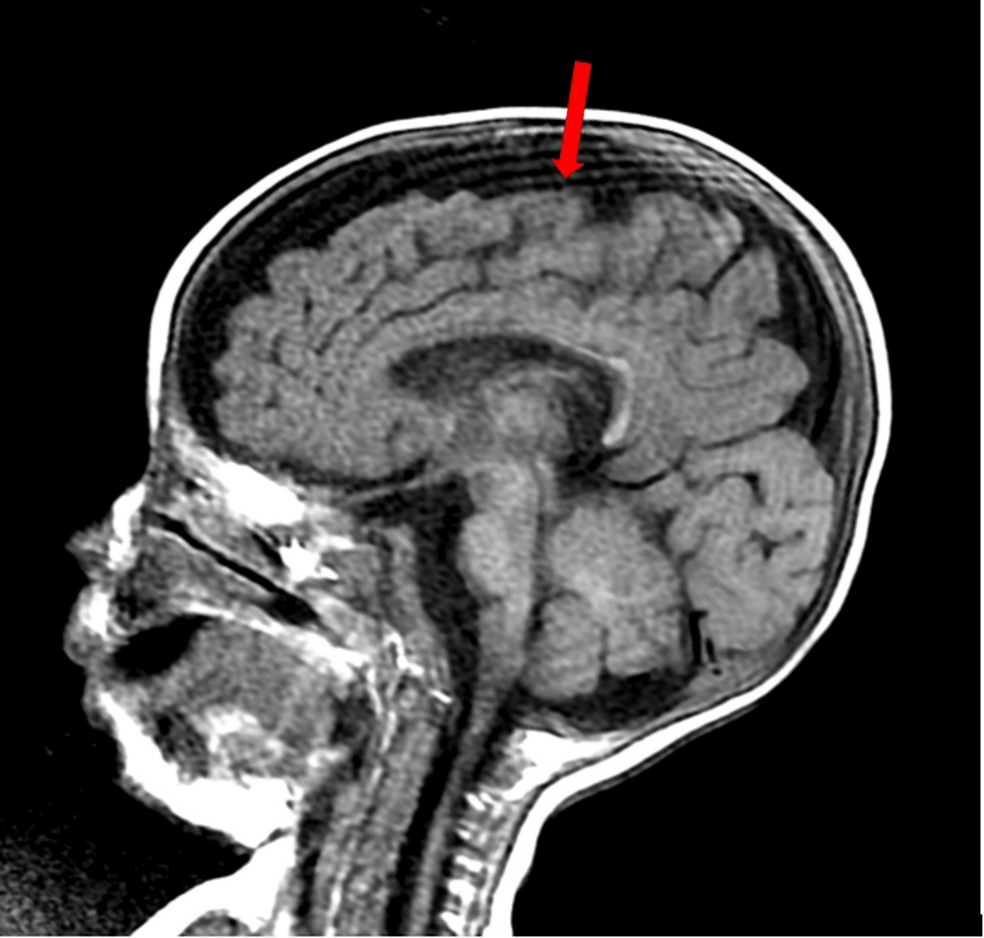
Sagittal slice of a brain MRI scan showing widening of the cortical sulci in relation to cortical atrophy

**Figure 3:**
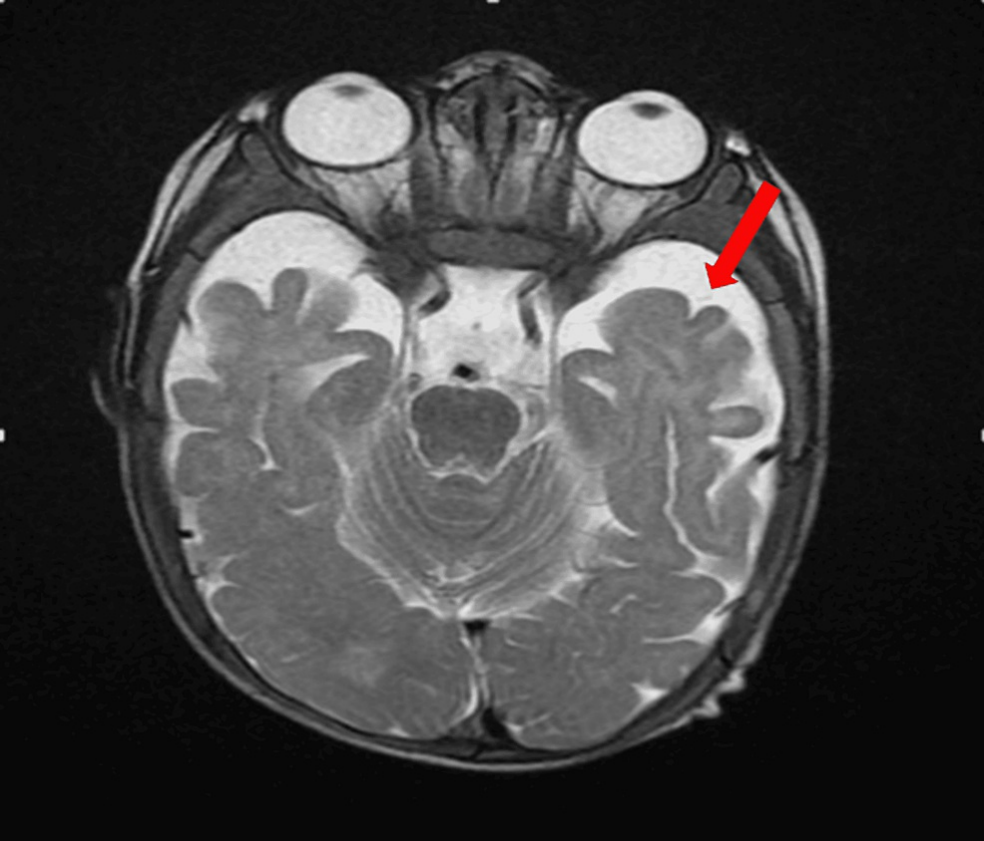
Axial section of a brain MRI scan showing widening of the cortical sulci in relation to cortical atrophy.

A bone marrow aspiration was performed to rule out a neoplastic origin (Figure 1). It revealed numerous megakaryocytes, sometimes dysplastic, with cellular gigantism: megaloblasts and giant metamyelocytes, a cytological appearance pointing to an anti-megaloblastic vitamin deficiency (vitamins B12 and B9). A cerebral MRI revealed bilateral frontoparietal cortical atrophy, with enlargement of the lateral ventricles and cortical sulci (Figure 2, Figure 3). Substitutive treatment with vitamin B12 supplementation was initiated with intramuscular injections of 1 mg/day for eight days, then 1 mg/week for one month, then 1 mg/month. Only one presentation of hydroxycobalamin is available on the Moroccan market, in the form of ampoules containing 5000 ug.

The infant showed significant improvement in clinical, hematological, and neurological aspects right from the start of the treatment. Follow-up was conducted regularly, initially weekly and then monthly, to assess the child's clinical condition and response to treatment. This follow-up was consistently maintained for several months after correcting the vitamin B12 deficiency, ensuring adequate vitamin B12 levels and normal neurological development and growth in the child.

## Discussion

Vitamin B12 deficiency is an uncommon pathology in pediatric patients, particularly in infants [2,7]. It was first characterized and individualized by Jadhav et al. in 1962 in six Indian infants, and subsequently reprocessed several times [7,8].

Vitamin B12, also known as cobalamin, is one of the eight B vitamins derived from foods of animal origin, found mainly in fish, meat, and dairy products [5]. Daily requirements for this vitamin are estimated at 1-2 picograms in children [9]. The supply of this vitamin to the fetus is transplacental, and in the newborn, the stock is present in the liver and is estimated at 25 ug. This quantity present in breast milk is parallel to that present in serum, and is supposed to be sufficient until six to eight months, or even the end of the first year [5,7,9]. However, endogenous reserves can be considerably reduced if the mother is deficient, as in our patient's case, which explains the clinical symptoms observed at four months of age. The literature reports that, among 100 or so vitamin B12-deficient infants studied, around 64% were exclusively breastfed by vegetarian mothers, 24% had mothers with Biermer's disease, and the remainder were linked to digestive pathologies or general deficiencies [7]. The mechanisms responsible for this condition are explained either by insufficient intake, absorption anomalies, or congenital transport or metabolism anomalies [2].

In the intestine, this vitamin binds to the intrinsic factor produced by gastric parietal cells. Once absorbed by the enterocyte, it is released into the portal circulation and transported to the tissues by transcobalamin II, a second transporter [5]. Vitamin B12 is a water-soluble vitamin. In its reduced form (mono- or divalent cobalt), it acts as a cofactor with vitamin B6 and folates, firstly to convert homocysteine to methionine by methylation in the cytoplasm. Secondly, via 5'deoxy-adenosylcobalamin, it converts methylmalonyl coenzyme A to succinyl coenzyme A in the mitochondria. These two chemical reactions reduce two potentially toxic substances: homocysteine, which can cause vascular endothelial damage, and methylmalonate, which contributes to metabolic acidosis [2,5]. This mechanism explains why our patient had a vitamin B12 deficiency and increased homocysteine.

Vitamin B12 plays an essential role in cellular metabolism, enabling the multiplication of rapidly-renewing cells such as hematopoietic cells by inducing premature cell death of erythroid precursors in the bone marrow, known as “intramedullary abortion” [5]. On the blood count, this manifests itself as frank macrocytic anemia (GMV greater than 110 fl), normochromic and aregenerative, with medullary megaloblastosis, giving a “blue marrow” appearance [9]. Neurological damage appears to be more severe in infants, with a cerebral rather than medullary expression, and the long-term prognosis is poorly understood, with several cases of psychomotor sequelae [5].

The clinical manifestations of vitamin B12 deficiency in infants are polymorphous, encompassing a digestive picture associating feeding difficulties, regurgitation, constipation, and weight and statural growth retardation explained by anorexia and vomiting [7,9], a neurological picture associating hypotonia, developmental delay or psychomotor regression of smiling and babbling as in our patient's case, abnormal movements, epileptic seizures, cerebral atrophy, or delayed myelination [10].

Biologically, blood counts are used to identify macrocytic, aregenerative anemia, with mean corpuscular volume (MCV) well above 100 fL, depending on age. Sometimes macrocytosis is masked by associated iron deficiency, inflammatory syndrome, or hemoglobinopathy [2,5], resulting in normocytic anemia. This anemia is sometimes absent in children and may be masked by folate excess [5]. Moderate leukopenia between 3,000 and 6,000 elements/mm^3^ is often observed and/or thrombocytopenia around 100,000/mm^3^. Pancytopenia is found more rarely [2]. Megaloblastosis is inconstant [2]. Serum vitamin B12 levels are low, as are homocysteine accumulation and plasma and urinary methylmalonic acid, confirming vitamin B12 deficiency; folate levels are normal, as are anti-factor antibodies and/or a Schilling test [7].

Treatment is based on vitamin B12 supplementation with intramuscular injections of 1 mg/day for eight days, then 1 mg/week for one month, then 1 mg/month. It should be noted that the only hydroxycobalamin available in Morocco is in the form of 5 mg ampoules [2,5]. Some studies describe the appearance of abnormal movements at the start of treatment [5].

The long-term prognosis remains uncertain and poorly understood, with many cases of sequential psychomotor impairment, especially if the diagnosis is made after the age of one. Hence, it is important to prevent vitamin B12 deficiency in pregnant women and nursing mothers on a strict vegetarian diet, or with Biermer disease.

## Conclusions

Vitamin B12 deficiency should be suspected in infants showing psychomotor regression, associated with anemia and stagnation of weight and height. Our patient presented with an identical clinically evident presentation and showed significant improvement in clinical, hematological, and neurological aspects from the start of the treatment. Follow-up was conducted regularly and consistently maintained for several months to ensure normal neurological development and growth of the child.

Screening of pregnant women and/or newborns is important, supported by the existence of a simple, effective intramuscular, or sublingual treatment that ensures improvement and reversibility of neurological damage.
